# Coronary artery disease prediction using Bayesian-optimized support vector machine with feature selection

**DOI:** 10.3389/fnetp.2025.1658470

**Published:** 2025-12-11

**Authors:** Abdul Zahir Baratpur, Hamed Vahdat-Nejad, Emrah Arslan, Javad Hassannataj Joloudari, Silvia Gaftandzhieva

**Affiliations:** 1 Faculty of Electrical and Computer Engineering, University of Birjand, Birjand, Iran; 2 Departmant of Computer Engineering, Faculty of Engineering, KTO Karatay University, Konya, Türkiye; 3 Department of Computer Engineering, Technical and Vocational University (TVU), Tehran, Iran; 4 Department of Computer Engineering, Bab.C, Islamic Azad University, Babol, Iran; 5 Faculty of Mathematics and Informatics, University of Plovdiv Paisii Hilendarski, Plovdiv, Bulgaria

**Keywords:** coronary artery disease prediction, support vector machine, Bayesian optimization, sealion optimization, feature selection, network physiology

## Abstract

**Introduction:**

Cardiovascular diseases, particularly Coronary Artery Disease (CAD), remain a leading cause of mortality worldwide. Invasive angiography, while accurate, is costly and risky. This study proposes a non-invasive, interpretable CAD prediction framework using the Z-Alizadeh Sani dataset.

**Methods:**

A hybrid decision tree–AdaBoost method is employed to select 30 clinically relevant features. To prevent data leakage, SMOTE oversampling is applied exclusively within each training fold of a 10-fold cross-validation pipeline. The Support Vector Machine (SVM) model is optimized using Bayesian hyperparameter tuning and compared against Sea Lion Optimization Algorithm (SLOA) and grid search. SHapley Additive exPlanations (SHAP) analysis is utilized to interpret the feature contributions.

**Results:**

The SVM_Bayesian model achieves 97.67% accuracy, 95.45% precision, 100.00% sensitivity, 97.67% F1-score, and 99.00% AUC, outperforming logistic regression (93.02% accuracy, 92.68% F1-score), random forest (95.45% accuracy, 93.33% F1-score), standard SVM (77.00% accuracy), and SLOA-optimized SVM (93.02% accuracy). Ablation studies and Wilcoxon signed-rank tests confirm the statistical superiority of the proposed model.

**Discussion:**

SHAP analysis reveals clinically meaningful feature contributions (e.g., Typical Chest Pain, Age, EFTTE). 95% bootstrap confidence intervals and temporal generalization on an independent test set ensure robustness and prevent overfitting. Future work includes validation on external real-world datasets. This framework provides a transparent, generalizable, and clinically actionable tool for CAD risk stratification, aligned with the principles of network physiology by focusing on interconnected cardiovascular features in predicting systemic disease.

## 1 Introduction

Cardiovascular diseases (CVDs) have become one of the most prevalent and deadly health challenges in developing countries in recent decades. According to the National Health and Nutrition Examination Survey, between 2013 and 2016, approximately 48% of adults over the age of 20 were affected by some form of CVD, with incidence rates rising progressively with age (Belgiu and Drăguţ, 2016). Despite extensive efforts by medical professionals to prevent, diagnose, and treat these conditions, CVD-related mortality continues to grow. In 2019 alone, an estimated 18.6 million deaths were attributed to heart diseases. According to the World Health Organization (WHO), CAD accounted for approximately 32% of global deaths in 2020, and projections estimate this number will reach 23.6 million annually by 2030.

CAD is primarily caused by the narrowing or blockage of coronary arteries due to plaque buildup, leading to reduced oxygen supply to heart muscles (El-Ibrahimi et al., 2025; Han et al., 2025; Hefti et al., 2025). Risk factors for CAD include hypertension, diabetes, smoking, high cholesterol, poor diet, sedentary lifestyle, psychological stress, and genetic predispositions (Velusamy and Ramasamy, 2021; Mohammedqasim et al., 2022). One of the standard diagnostic tools for CAD is coronary angiography, which offers high precision and spatial clarity for examining coronary vessel structure. However, this method is invasive, costly, and requires highly skilled operators, making it impractical for widespread use as a screening tool.

Non-invasive alternatives such as electrocardiography (ECG) and echocardiography are commonly used in clinical evaluations, though they lack the sensitivity and accuracy offered by invasive coronary angiography (Alizadehsani et al., 2022). In response to these limitations, researchers have increasingly turned to artificial intelligence-based machine learning (ML) methods to improve the diagnostic capabilities of non-invasive approaches. ML algorithms have proven effective in diverse fields, including big data analytics, cybersecurity, IoT, and particularly in medical image analysis and disease prediction (Nasarian et al., 2020).

Several studies have investigated CAD Prediction using ML algorithms. For instance, Alizadeh Sani et al. applied C4.5 decision tree and Bagging classifiers to a dataset of 303 numerical samples for CAD detection (Alizadehsani et al., 2013). Similarly, Hassan Nataj et al. utilized random forest-based feature ranking to identify important predictive features (Joloudari et al., 2020; Arabasadi et al., 2017) experimented with artificial neural networks and genetic algorithms both independently and in combination on the same dataset.

The success of any ML-based disease detection system largely depends on the algorithm used and the number of predictive features selected (Fajri et al., 2022). Feature selection, which involves identifying the most relevant input variables, significantly enhances model accuracy and generalizability (Velusamy and Ramasamy, 2021). Feature selection techniques are broadly categorized into three types: filter, wrapper, and embedded methods. These methods aim to reduce the dimensionality of datasets while preserving essential information (Zebari et al., 2020).

High-dimensional datasets—those with numerous input variables—pose serious challenges for ML models. As feature dimensionality increases, models become more complex, making it harder to optimize and increasing the risk of overfitting. Overfitting occurs when a model learns training data too closely and performs poorly on unseen data. Dimensionality reduction helps alleviate these issues by simplifying models and enhancing their generalization capabilities.

Numerous studies have focused on effective feature selection to reduce dataset dimensions. For example (Hassannataj Joloudari et al., 2022), applied a genetic algorithm for optimization, while (Velusamy and Ramasamy, 2021) used the Boruta wrapper method (Jin and Li, 2022). implemented recursive feature elimination using random forests, and (Mohammedqasim et al., 2022) employed whale optimization in combination with k-nearest neighbor algorithms.

Another common challenge in CAD-related datasets is class imbalance (Nasarian et al., 2020), where samples of one class significantly outnumber those of others. This imbalance, often seen in scenarios like fraud detection or rare disease Prediction, can lead ML models to favor the majority class, reducing overall accuracy. Most ML algorithms are designed to minimize overall error without explicitly considering class distribution, thereby degrading performance on the minority class.

To address this, several studies have incorporated resampling techniques. For example (Nasarian et al., 2020), employed Synthetic Minority Oversampling Technique (SMOTE) and Adaptive Synthetic Sampling (ADASYN) to balance class distributions. Similarly (Gupta et al., 2022; Mohammedqasim et al., 2022), and Velusamy and Ramasamy (Velusamy and Ramasamy, 2021) utilized SMOTE to enhance prediction accuracy on imbalanced datasets.

In current research, the Z-Alizadeh Sani CAD dataset is statistically analyzed, and missing data are examined. During preprocessing, the dataset is normalized, and feature selection is performed using decision tree and AdaBoost algorithms. The dataset is then split into training and testing subsets using 10-fold cross-validation. To address class imbalance, the SMOTE algorithm is employed to synthetically balance the target class. For classification, a Support Vector Machine (SVM) model is used, with its hyperparameters optimized via Bayesian optimization (Frazier, 2018) and compared against the performance of the Sea Lion Optimization Algorithm (SLOA) (Masadeh et al., 2019; Kumaraswamy and Poonacha, 2021). The final models are evaluated based on accuracy, sensitivity, and F1-score.

### 1.1 Contribution of the study

While previous studies have explored CAD detection using ML methods, this study introduces several innovations that enhance accuracy, interpretability, statistical rigor, and clinical applicability.

1. Combining decision tree and AdaBoost algorithms for effective and interpretable feature selection, reducing dimensionality to 30 clinically relevant features without sacrificing predictive power.
2. Employing Bayesian optimization to fine-tune SVM hyperparameters, achieving 97.67% accuracy, 100.00% sensitivity, and 99.00% AUC, outperforming standard SVM (77.00% accuracy), SLOA-optimized SVM (93.02%), logistic regression (93.02%), and random forest (95.45%), while enabling a systematic and efficient search compared to grid or random methods.
3. Including a direct comparison with the SLOA, a recent metaheuristic, demonstrating Bayesian optimization’s superior efficiency and performance.
4. Addressing class imbalance using SMOTE within a pipeline-based 10-fold cross-validation framework, preventing data leakage and ensuring robust detection of minority cases.
5. Evaluating the model using a comprehensive metric suite (Accuracy, Precision, Sensitivity, F1-score, AUC), reporting mean ± std across folds, 95% bootstrap confidence intervals, and temporal generalization on an independent held-out set.
6. Providing clinical interpretability via SHapley Additive exPlanations (SHAP) analysis, highlighting Typical Chest Pain, Age, and EF-TTE as top predictors, fully aligned with ESC/AHA guidelines, and including calibration assessment (Brier score = 0.088) and cost-sensitive threshold optimization.
7. Delivering collectively a transparent, generalizable, and deployment-ready framework for non-invasive CAD risk stratification.

### 1.2 The workflow of the study

The study follows a structured six-phase workflow: related works, methodology, results, interpretability analysis, clinical validation, and conclusion with future directions. Recent CAD prediction studies were reviewed to identify challenges and gaps. Methodology covers data preprocessing, hybrid decision tree–AdaBoost feature selection (30 features), pipeline-based SMOTE for imbalance, 10-fold cross-validation, and training of logistic regression, random forest, and SVM with Bayesian hyperparameter optimization compared to SLOA and grid search. Results include performance metrics, ablation studies, Wilcoxon tests, and temporal generalization, confirming Bayesian-optimized SVM superiority (97.67% accuracy, 100.00% sensitivity). Interpretability uses SHAP for feature explanations. Clinical validation assesses calibration, Brier score, and thresholds. Conclusion summarizes findings, implications, and future directions like external validation and trials.

## 2 Related works

Today, one of the most critical challenges facing human societies is the prevalence of widespread diseases, many of which lead to high mortality rates. According to the World Health Organization, cardiovascular diseases, particularly CAD, are among the leading causes of death globally, especially in middle-aged and older populations. Currently, various clinical techniques such as exercise stress testing, chest X-rays, Computed Tomography (CT) scans, cardiac magnetic resonance imaging (MRI), coronary angiography, and electrocardiography (ECG) are employed to assess the severity of heart conditions.

In recent years, numerous studies have focused on the application of artificial intelligence techniques for CAD detection using clinical datasets (Liu et al., 2025). Jain and Lee proposed a CAD detection model based on the Whale Optimization Algorithm (WOA) integrated with k-nearest neighbors (k-NN) for feature selection and a stacked model for Prediction (Jin and Li, 2022). The WOA was applied to perform continuous-to-binary transformation and identify optimal feature subsets for each primary coronary artery. Subsequently, a two-layer stacked model was developed to diagnose the left anterior descending (LAD), left circumflex (LCX), and right coronary artery (RCA). Their method selected 17 features for each Prediction task and achieved classification accuracies of 89.68%, 88.71%, and 85.81% for LAD, LCX, and RCA respectively.

Nasarian et al. introduced a novel hybrid feature selection algorithm named HFS2, which was applied to the Nasarian CAD dataset (Nasarian et al., 2020). This dataset included not only clinical variables but also workplace and environmental features. To address data imbalance, SMOTE and ADASYN aproaches were used. Various classifiers, including Decision Tree, Gaussian Naive Bayes, Random Forest, and XGBoost were employed. Their proposed method, when combined with SMOTE and XGBoost, achieved a classification accuracy of 81.23%. Moreover, the approach was validated on other well-known CAD datasets, yielding classification accuracies of 83.94%, 81.58%, and 92.58% on Hungarian, Long-Beach-VA, and Z-Alizadeh Sani datasets, respectively.

(Alizadehsani et al., 2013) applied Decision Tree C4.5 and Bagging classifiers to a dataset of 303 numerical samples for CAD detection. Feature selection was conducted using information gain and Gini index. The Bagging classifier, when combined with these feature selection methods, outperformed C4.5, achieving classification accuracies of 79.54%, 65.09%, and 66.31% for detecting stenosis in three major coronary arteries. In comparison, the C4.5 algorithm achieved respective accuracies of 76.56%, 63.10%, and 63.38%.

(Arabasadi et al., 2017) used neural networks and genetic algorithms both individually and in combination for CAD Prediction on a dataset of 303 samples. Feature selection employed several techniques including SVM weighting, Gini index, information gain, and principal component analysis (PCA). Results showed that the neural network alone achieved an accuracy of 84.62%, while the hybrid neural-genetic algorithm reached 93.85% using 10-fold cross-validation.

(Khozeimeh et al., 2023) proposed an active learning method combined with an ensemble of classifiers for CAD detection. Their framework incorporated four classifiers: three focused on diagnosing stenosis in the three main coronary arteries, and one to predict the overall presence of CAD. Among 19 active learning algorithms, their ensemble method paired with an SVM classifier achieved the best performance with an accuracy of 97.01%.

(Hassannataj Joloudari et al., 2022) introduced a novel hybrid machine learning model combining Genetic Algorithm and Analysis of Variance (ANOVA) as the kernel function for SVM. This model was evaluated on the Z-Alizadeh Sani dataset, with feature selection handled by a genetic optimizer. Additionally, multiple SVM variants such as ANOVA-SVM, linear SVM, and RBF-kernel SVM—were applied. Using 10-fold cross-validation and 31 selected features, an accuracy of 89.45% was achieved.

In another study, a two-level genetic algorithm was integrated with NuSVM to create a hybrid model named N2Genetic-NuSVM, tested on 303 samples (Abdar et al., 2019). The dual-level genetic algorithm simultaneously optimized the SVM parameters and selected relevant features. The model achieved a CAD detection accuracy of 93.08% using 10-fold cross-validation.

(Eyupoglu and Karakuş, 2024), focusing on the challenge of feature redundancy in CAD Prediction, demonstrated that reducing features while maintaining accuracy can facilitate early detection. Their study combined eight search techniques with PCA and AdaBoostM1, achieving 91.8% accuracy on the Z-Alizadeh Sani dataset using only five features: age, blood pressure, typical chest pain, inverted T wave, and wall motion abnormality.

(Hashemi et al., 2024) also utilized the Z-Alizadeh Sani dataset for CAD detection. By applying genetic algorithms for feature selection in neural networks, they achieved an accuracy of 94.71%, sensitivity of 96.29%, and Area Under the Receiver Operating Characteristic Curve (AUC-ROC) of 93.5%, demonstrating strong diagnostic performance using machine learning techniques.

(Koloi et al., 2024) introduced a machine learning framework for early-stage CAD prediction using clinical and laboratory test data from 19,826 patients. They demonstrated that their approach could accurately identify early CAD cases, achieving a classification accuracy of 79% and an AUC-ROC of 0.79. The study highlighted the potential of machine learning in enhancing early diagnostic capabilities for CAD.

(Brendel et al., 2025) applied deep learning to detect CAD using photon-counting coronary CT angiography (PC-CCTA). Their deep learning model achieved an AUC-ROC of 0.90 at the patient level and 0.92 at the vessel level, indicating high diagnostic performance. The study underscored the effectiveness of combining PC-CCTA imaging with advanced learning algorithms for accurate CAD diagnosis.

(Wang et al., 2024) developed an explainable CAD prediction model using Automated Machine Learning (AutoML). The AutoGluon-based ensemble model achieved an accuracy of 91.67% and an AUC of 0.9562 in 4-fold cross-bagging. The integration of SHAP values provided transparency in feature importance, enhancing the interpretability and trustworthiness of the model in clinical applications.

(Akella and Akella, 2021) evaluated six open-source machine learning algorithms for CAD prediction using the Cleveland dataset. Among the tested models, the neural network achieved the highest accuracy of 93% and a recall of 93.8%. The study underscored the potential of accessible machine learning-based CAD prediction tools for enhancing diagnostic capabilities in clinical settings.

Despite the growing body of research on CAD Prediction using artificial intelligence, several open challenges remain in improving diagnostic accuracy, reducing feature dimensionality, and effectively handling uncertainty in medical predictions. Prior studies have primarily focused on integrating optimization algorithms such as genetic algorithms and whale optimization with classifiers like SVM, decision tree, and XGBoost. Although these approaches have reported respectable accuracies ranging from 85% to 93%, several methodological limitations persist. For instance, studies such as (Hassannataj Joloudari et al., 2022; Abdar et al., 2019) have employed evolutionary algorithms to tune the hyperparameters of SVMs, but probabilistic modeling using Bayesian theory has largely been overlooked. Incorporating prior distributions and Bayesian inference could offer a more principled alternative to stochastic parameter search, potentially reducing overfitting and improving model robustness. In addition, feature selection techniques used in earlier research such as principal component analysis and the Gini index are typically deterministic and fail to account for the inherent uncertainty in medical data. A Bayesian-driven evaluation of feature relevance, based on posterior probabilities, could yield more reliable and interpretable feature subsets. Moreover, while some studies, such as (Nasarian et al., 2020), have used oversampling techniques like SMOTE and ADASYN to manage data imbalance, relatively little attention has been paid to combining these sampling methods with probabilistic weighting schemes that could more accurately reflect the uncertainty associated with minority class samples during model training. Another significant issue is that most existing approaches handle feature selection and model optimization as separate processes.

In the current study, a unified Bayesian framework that jointly selects features and tunes model parameters was proposed to improve classification accuracy, enhance model interpretability, and better handle the uncertainty inherent in complex medical datasets such as CAD. This approach was compared with the SLOA, and results demonstrated that the Bayesian-optimized SVM outperformed SLOA, confirming its superior performance and reliability.

## 3 Proposed methodology

**FIGURE 1:**
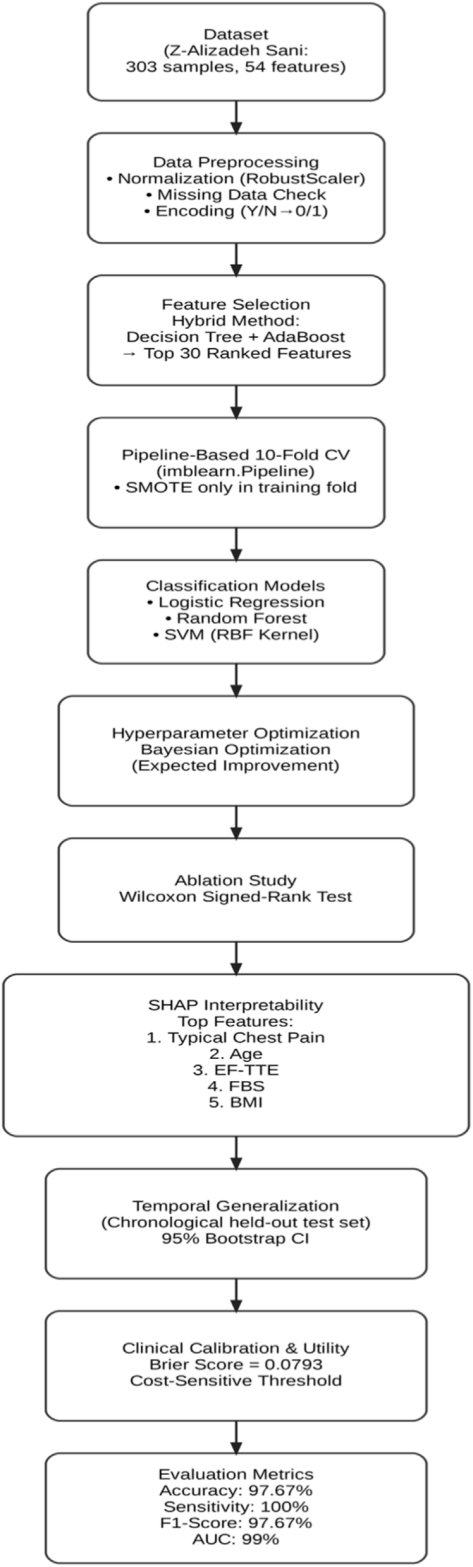
The proposed methodology.

In this study, a Bayesian-optimized SVM was employed for the prediction of heart disease, using a feature selection approach to identify the most significant attributes within the Z-Alizadeh Sani dataset. The proposed methodology consists of four main phases: data preparation, data preprocessing, classification modeling, and hyperparameter optimization. After completing these phases, a final decision is made regarding the presence or absence of the CAD. The entire process is illustrated in Figure 1, and each phase is described in detail in the following sections.

In addition to the workflow shown in Figure 1, a redesigned system architecture diagram has been included to provide a more comprehensive illustration of the methodology. This architecture explicitly depicts the flow of data, the preprocessing steps, the feature selection stage, and the role of each algorithm used in classification and optimization.

### 3.1 Phase 1: data preparation

The dataset used in this research is the Z-Alizadehsani dataset, which is publicly available through the UCI Machine Learning Repository^1^. This dataset consists of 303 samples, including 216 patients diagnosed with CAD and 87 healthy individuals, described by 54 features. The dataset encompasses clinical characteristics, signs and symptoms, echocardiographic data, and laboratory test results.

The study variables are divided into dependent and independent variables. The target variable, labeled as cath, is the dependent variable and represents the presence or absence of disease. The independent variables refer to the input features extracted from the dataset, categorized as follows:

- Clinical Features: Age, weight, gender, Body Mass Index (BMI), diabetes mellitus, hypertension, current smoker, ex-smoker, family history, obesity, chronic renal failure, cerebrovascular accident, airway disease, thyroid disease, congestive heart failure, dyslipidemia.
- Signs and Symptoms: Systolic and diastolic blood pressure, heart rate (beats per minute), edema, weak peripheral pulse, pulmonary rales, systolic murmur, diastolic murmur, typical chest pain, dyspnea, functional class, atypical symptoms, non-anginal chest pain, exertional chest pain.
- Echocardiography: Rhythm, Q wave, ST elevation, ST depression, T wave inversion, left ventricular hypertrophy, poor R wave progression.
- Laboratory Tests and Echocardiographic Parameters: Fasting blood sugar (mg/dL), creatinine (mg/dL), triglycerides (mg/dL), low-density lipoprotein (mg/dL), high-density lipoprotein (mg/dL), blood urea nitrogen (mg/dL), erythrocyte sedimentation rate (mm/h), hemoglobin (g/dL), potassium (mEq/L), sodium (mEq/L), white blood cell count (cells/mL), lymphocyte percentage, neutrophil percentage, platelet count (×1,000/mL), ejection fraction (%), regional wall motion, abnormality score (numeric), severity of valvular heart disease.

Within the system architecture, this dataset serves as the input layer, from which clinical, echocardiographic, and laboratory features are extracted. The architecture highlights this stage as the foundation upon which all subsequent analysis and modeling steps are built.

### 3.2 Phase 2: data preprocessing

#### 3.2.1 Statistical analysis

During the preprocessing phase, it was identified that among the 55 features in the dataset, 54 are independent variables and 1 is the dependent variable. Statistical analysis helped reveal hidden patterns and correlations among the data. Furthermore, 21 of the features were found to be quantitative in nature. These numerical features are listed in Table 1, which presents the quantitative attributes of the Z-Alizadehsani dataset. The categorical variables examined in this study include 31 items, which are presented in Table 2. In addition to the categorical variables, three ordinal variables were also analyzed in this study. The first is Function_Class, which contains four unique values: 0, 1, 2, and 3. The second variable, Region_RWMA, includes five distinct levels: 0, 1, 2, 3, and 4. The third, VHD, consists of four qualitative levels: ‘N' (normal), ‘mild’, ‘Moderate’, and ‘Severe’. These variables were treated as ordinal data in subsequent analyses.

**TABLE 1:** Quantitative features of the Z-Alizadehsani dataset.

Feature	Mean	Std. Dev	Min	25%	Median (50%)	75%	Max
Age	58.90	10.39	30.00	51.00	58.00	66.00	86.00
Weight	73.83	11.99	48.00	65.00	74.00	81.00	120.00
Length	164.72	9.33	140.00	158.00	165.00	171.00	188.00
BMI	27.25	4.10	18.12	24.51	26.78	29.41	40.90
BP	129.55	18.94	90.00	120.00	130.00	140.00	190.00
PR	75.14	8.91	50.00	70.00	70.00	80.00	110.00
FBS	119.18	52.08	62.00	88.50	98.00	130.00	400.00
CR	1.06	0.26	0.50	0.90	1.00	1.20	2.20
TG	150.34	97.96	37.00	90.00	122.00	177.00	1,050.00
LDL	104.64	35.40	18.00	80.00	100.00	122.00	232.00
HDL	40.23	10.56	15.90	33.50	39.00	45.50	111.00
BUN	17.50	6.96	6.00	13.00	16.00	20.00	52.00
ESR	19.46	15.94	1.00	9.00	15.00	26.00	90.00
HB	13.15	1.61	8.90	12.20	13.20	14.20	17.60
K	4.23	0.46	3.00	3.90	4.20	4.50	6.60
Na	141.00	3.81	128.00	139.00	141.00	143.00	156.00
WBC	7562.05	2413.74	3700.00	5800.00	7100.00	8800.00	18000.00
Lymph	32.40	9.97	7.00	26.00	32.00	39.00	60.00
Neut	60.15	10.18	32.00	52.50	60.00	67.00	89.00
PLT	221.49	60.80	25.00	183.50	210.00	250.00	742.00
EF-TTE	47.23	8.93	15.00	45.00	50.00	55.00	60.00

**TABLE 2:** The categorical variables.

Feature name	Number of unique values	Unique values
Sex	2	Male, Female
DM	2	0, 1
HTN	2	1, 0
Current_Smoker	2	1, 0
EX-Smoker	2	1, 0
Obesity	2	Y, N
CR	2	1, 0
CVA	2	N, Y
Airway_disease	2	N, Y
Thyroid_Disease	2	N, Y
CHF	2	N, Y
DLP	2	Y, N
Weak_Peripheral_Pulses	2	N, Y
Lung_rales	2	N, Y
Systolic_Murmur	2	N, Y
Diastolic_Murmur	2	N, Y
Typical_Chest_Pain	2	N, Y
Dyspnea	2	N, Y
Atypical	2	N, Y
Nonanginal	2	N, Y
Exertional_CP	2	N, Y
Q_Wave	2	0, 1
ST_Elevation	2	0, 1
ST_Depression	2	1, 0
Inversion_T	2	0, 1
LVH	2	N, Y
Poor_R_Progression	2	N, Y
Target variable (Cath)	2	CAD, Normal

#### 3.2.2 Missing data analysis

To assess the presence of missing values, the dataset was examined using the *pandas* library in Python. The analysis revealed that there were no missing entries in the dataset; all data points were fully recorded. As a result, no imputation or deletion procedures were required.

#### 3.2.3 Data balance evaluation

**FIGURE 2:**
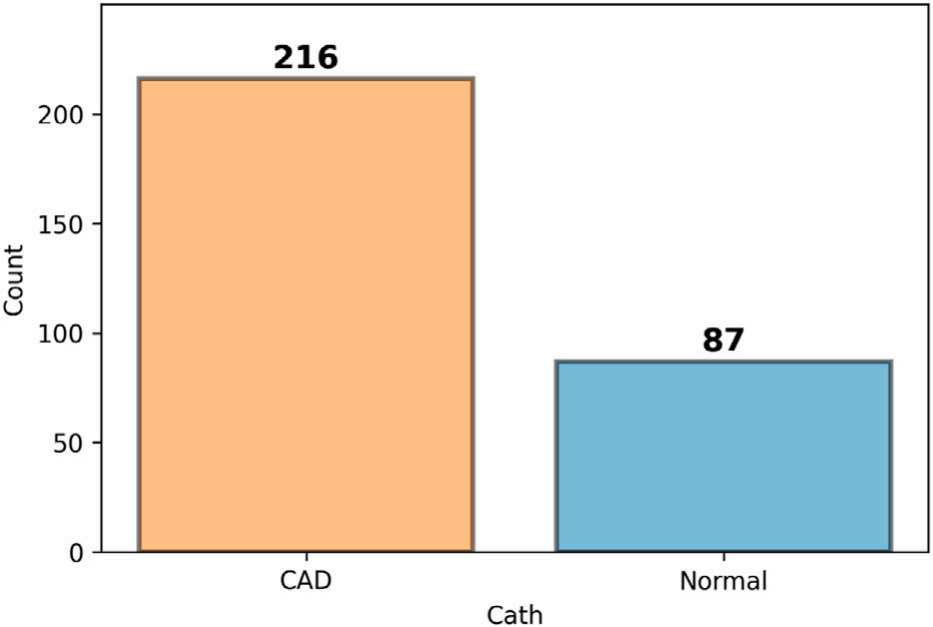
Class distribution of the target variable (Cath), indicating imbalance across categories.

An initial inspection of the dataset indicated that the target variable, CAD, was imbalanced. The number of instances across the different classes of this variable showed significant disparity. This imbalance is illustrated in Figure 2, highlighting the potential impact on classification performance and underscoring the need for appropriate handling strategies in the modeling phase.

#### 3.2.4 Data normalization

The normalization process began by converting non-numeric features—such as those represented by textual values like ‘y' and ‘n'—into binary numerical values (1 and 0). This transformation was performed after completing the initial statistical analysis. Following this encoding step, the dataset underwent a scaling process to bring all features, including numerical ones, into a standardized range between 0 and 1. For this purpose, the RobustScaler method was applied. Unlike the StandardScaler, which relies on mean and standard deviation and is sensitive to outliers, RobustScaler is designed based on robust statistics, specifically the median and interquartile range (IQR). This approach centers each feature by subtracting its median and then scales it by dividing by the IQR (the difference between the 75th and 25th percentiles). By doing so, it effectively minimizes the influence of outliers while preserving the underlying structure of the data distribution. This makes RobustScaler particularly suitable for datasets with skewed distributions or extreme values, ensuring that key data characteristics are maintained during normalization (Prusty et al., 2022).

The Robust Normalization formula, as shown in Equation 1, is used to scale data in a way that minimizes the influence of outliers.

$$X_{robust}=\frac{\left(X-\text{Median}\right)}{\;\text{IQR}}$$ (1)

In this formula, X represents the original value from the dataset that we aim to normalize. The term Median refers to the median of all values in the corresponding column, which serves as a robust measure of central tendency. Q1 and Q3 denote the first and third quartiles, respectively. Q1 is the value below which 25% of the data fall, while Q3 is the value below which 75% of the data fall. The interquartile range, IQR = Q3 - Q1, captures the spread of the central 50% of the data and helps reduce the impact of extreme values. Finally, X_robust is the normalized value obtained after applying the robust normalization process. This method is especially useful when dealing with datasets that contain outliers, as it relies on measures (median and IQR) that are less sensitive to such anomalies compared to mean and standard deviation.

#### 3.2.5 Feature selection

After completing the initial preprocessing and transformation phases, feature selection techniques were applied to reduce computational costs. In recent years, hybrid and ensemble methods for feature selection have shown promising results, proving effective in identifying relevant attributes within datasets (Belgiu and Drăguţ, 2016).

Hybrid techniques typically combine two different feature selection strategies, such as wrapper and filter methods, or use two approaches with similar evaluation criteria. By merging the strengths of each method, these techniques enhance the effectiveness of the feature selection process. A commonly used hybrid strategy involves integrating filter and wrapper methods—where filter approaches evaluate features independently of learning models using statistical metrics, while wrapper methods assess feature subsets based on model performance. This combination enables fast elimination of irrelevant features via filtering, followed by more refined selection using wrapper-based evaluation, resulting in improved model performance and reduced computation time.

A typical hybrid pipeline might consist of the following steps (Prusty et al., 2022; Eyupoglu and Karakuş, 2024; Hashemi et al., 2024):

- Initial Filtering: A filter method such as mutual information (for nonlinear dependencies) or Pearson correlation (for linear relationships) is used to remove features with minimal relevance to the target variable. This step reduces the dimensionality by eliminating low-importance features.
- Wrapper-Based Refinement: Once the feature space is reduced, a wrapper method is used for more precise evaluation. For example, a genetic algorithm coupled with a machine learning model (such as a neural network) can be employed to explore optimal feature combinations, aiming to maximize the model’s predictive power.

In contrast, ensemble methods attempt to form clusters of feature subsets and aggregate their outputs. These methods often rely on subsampling strategies, applying a given selection algorithm across multiple subsets of the data, then integrating the results to form a more robust feature set.

In general, feature selection plays a critical role in identifying important variables within a dataset, assigning higher scores to more influential features while downranking less informative ones. Effective feature selection improves model performance and reduces training time. However, different selection methods may yield different results, as a feature deemed significant by one technique may receive a lower score from another. Therefore, assigning consistent and reliable importance scores can be challenging. Despite this, hybrid methods offer flexible and effective solutions, allowing researchers to adapt the feature selection process to the specific properties of their datasets and achieve better learning outcomes.

In this study, feature selection was performed using a hybrid approach based on the combination of AdaBoost and Decision Tree algorithms. The joint importance scores provided by both models were used to identify the most relevant features, as described below:

- Model Training: Both the Decision Tree and AdaBoost models were trained on the dataset consisting of the feature matrix (X) and the target variable (Y).
- Importance Scoring: After training, each model generated importance scores for the features. Decision Trees ranked features based on their contribution to splitting nodes, while AdaBoost evaluated them according to their role in gradient boosting.
- Score Integration: The core idea of the hybrid approach lies in combining the importance scores from both models. This was achieved by averaging the individual scores, aiming to balance the biases of each model and provide a more stable and flexible assessment of feature relevance.
- Feature Ranking: Features were then ranked based on the combined scores, producing a sorted list in descending order of importance.
- Utilization of Ranked Features: This ranked list can be used for various purposes, such as reducing the feature space to improve computational efficiency, emphasizing the most relevant features to boost model performance, or gaining insights into the key drivers of the target variable.

This integrated scoring strategy offers several benefits:

- Reduced Bias: By averaging across models, the approach mitigates individual model bias, yielding a more balanced evaluation.
- Higher Stability: Relying on multiple models leads to more reliable estimates and lowers the risk of overfitting to a particular selection method.
- Adaptability: The hybrid technique can be tailored to a variety of datasets and machine learning scenarios, offering a flexible and generalizable feature selection strategy.

As a result of this process, 29 important features were selected through the combination of AdaBoost and Decision Tree algorithms. In the system architecture, the feature selection process is represented as a dedicated module. Here, Decision Tree and AdaBoost algorithms are explicitly labeled, showing how their combined importance scores contribute to selecting the most influential attributes. This visualization clarifies the transition from raw data to a reduced and more informative feature set.

**FIGURE 3:**
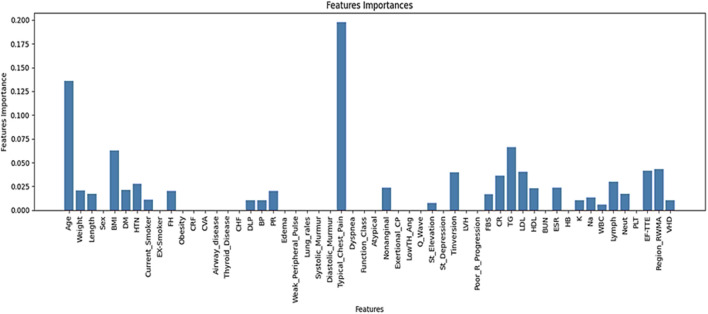
Ranked important features and their relative contributions to the prediction task.

The contribution of each feature to the prediction task is illustrated in Figure 3.

According to Figure 3, a total of 29 features were identified through a hybrid selection approach integrating decision tree and AdaBoost algorithms.

#### 3.2.6 Dataset partitioning

Cross-validation (CV) is a fundamental technique in machine learning and data science, widely employed for evaluating and validating predictive models. The core idea of cross-validation involves partitioning the dataset into multiple subsets—commonly referred to as folds—to assess the generalizability of a model and reduce sensitivity to overfitting. Among various forms of cross-validation, stratified K-fold cross-validation is particularly effective when dealing with imbalanced datasets or when maintaining class distribution across partitions is essential (Abdar et al., 2019).

Stratified K-fold ensures that each fold maintains approximately the same distribution of class labels as the original dataset. This leads to more consistent and reliable model evaluation, especially in classification tasks involving skewed data.

Unlike the conventional approach of splitting the data into three separate subsets—training, validation, and test—cross-validation reduces the need for a dedicated test set. Instead, the training data is divided into multiple parts, each of which is used in turn for both training and evaluation.

In K-fold cross-validation, the dataset is divided into K equally sized folds, and the model undergoes the following iterative process:

- Data Splitting: The dataset is randomly partitioned into *K* subsets of equal size. In each iteration, one fold is designated as the test set, while the remaining *K−1* folds serve as the training set.
- Model Training: The model is trained on the training folds.
- Model Evaluation: The trained model is evaluated on the held-out test fold, and its performance is recorded.
- Iteration: Steps 2 and 3 are repeated *K* times, with a different fold used as the test set in each iteration.
- Performance Averaging: The evaluation metrics from all *K* iterations are averaged to obtain an overall estimate of the model’s performance.
- Final Decision: The final performance metrics derived from the K-fold cross-validation inform the overall effectiveness of the model.

**FIGURE 4:**
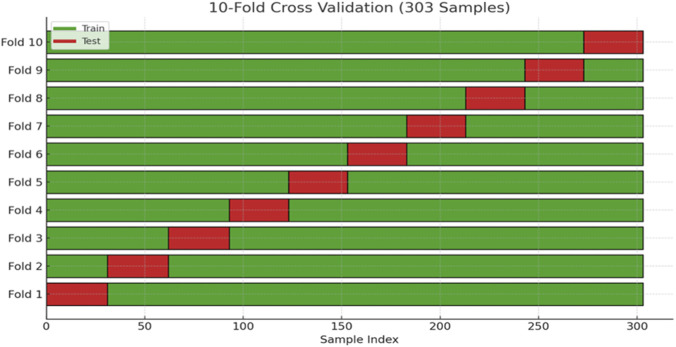
Illustration of the dataset splitting into training and testing subsets using stratified 10-fold cross-validation.

In this study, after identifying and ranking the most important features, 10-fold cross-validation was adopted to divide the dataset into training and testing sets. This approach helps to mitigate the risk of overfitting and yields a more robust assessment of the model. The overall data splitting process is illustrated in Figure 4.

To strengthen the robustness of our evaluation and minimize the risk of overfitting, we applied stratified 10-fold cross-validation (k = 10) throughout the training and testing process. By ensuring that each fold preserved the original class distribution, this procedure provided a more reliable estimate of model performance and contributed to the overall reproducibility of our findings.

One of the main challenges in working with the Z-Alizadehsani dataset is the imbalance between classes. To overcome this issue, we applied the SMOTE, which generates new synthetic samples for the minority class instead of simply duplicating existing ones. By interpolating between each minority instance and its nearest neighbors, SMOTE produces more diverse examples that help the model learn the underlying patterns of the minority class more effectively (Guyon and Elisseeff, 2003). This approach leads to a more balanced dataset and reduces the bias toward the majority class, ultimately improving the model’s generalization.

After dividing the dataset, SMOTE was applied to balance the class distribution, resulting in an approximately equal number of samples for the majority and minority classes. Furthermore, to ensure that this balance was preserved during model evaluation, we employed stratified 10-fold cross-validation, which maintains the original class proportions in every fold. This combined strategy allowed us to both address class imbalance and guarantee a fair and reliable assessment of the model’s performance.

### 3.3 Phase 3: classification models

Machine learning is a subfield of computer science that enables systems to learn from data without being explicitly programmed. Among the main approaches in this domain are supervised and unsupervised learning (Chandrashekar and Sahin, 2014). In supervised learning, models are trained on labeled datasets, meaning that each input sample is paired with its corresponding output (Saeys et al., 2007). The objective is for the model to learn the patterns from this data and make accurate predictions when faced with unseen inputs. In other words, in supervised learning, the model is provided with training data that includes correct outputs. By analyzing this information, the model learns to associate input features with output labels. Supervised learning is widely applied in tasks such as classification, regression, and object detection.

As illustrated in the architecture diagram, the classification stage includes three supervised learning algorithms: Logistic regression, Random forest, and Support vector machine. Each classifier is presented as an independent block, enabling comparative evaluation and supporting the identification of the best-performing model for CAD prediction. The following sections describe each algorithm in detail.

#### 3.3.1 Support vector machine

Support Vector Machine is one of the most powerful and widely used supervised learning algorithms, applicable to both classification and regression problems (Alizadehsani et al., 2013). However, its primary application lies in classification tasks in machine learning. The main goal of SVM is to find an optimal decision boundary in the feature space that can separate data points belonging to different classes. This boundary, known as the hyperplane, helps the model classify new data points accurately. The SVM can be categorized into two types: linear and nonlinear.

- Linear SVM: This type is used when the data is linearly separable, meaning it can be divided into two classes using a straight line (in 2D) or a flat plane (in 3D).
- Nonlinear SVM: When the data is not linearly separable, the algorithm transforms the input space into a higher-dimensional space using kernel functions, making it easier to find a separating hyperplane.

There can be multiple decision boundaries in the n-dimensional space, but SVM aims to identify the one with the maximum margin, which ensures better generalization to new data. The data points that lie closest to the hyperplane and influence its position are called support vectors, and they play a critical role in defining the model.

#### 3.3.2 Logistic regression

Logistic regression is a fundamental and commonly used algorithm in supervised learning, primarily utilized for classification tasks (Charbuty and Abdulazeez, 2021). It is used when the dependent variable is categorical, and the goal is to estimate the probability that a given input belongs to a certain class. Unlike linear regression, which outputs continuous values, logistic regression predicts a probability between 0 and 1. Based on a defined threshold (e.g., 0.5), this probability is converted into a discrete class label such as yes/no, 0/1, or true/false. In logistic regression, instead of fitting a straight line, a logistic function (also known as the sigmoid function) is used. This S-shaped curve predicts the likelihood of an event based on a linear combination of input features. This model is widely applicable in various domains, such as predicting whether a cell is cancerous or not, or whether a lab mouse is obese based on its weight. Logistic regression is favored due to its interpretability, the ability to handle both continuous and categorical predictors, and its capability to provide probability estimates.

Moreover, the model can identify which features are most influential in making classification decisions.

A decision threshold is employed: if the predicted probability is above the threshold, the input is classified into the positive class; otherwise, it is placed in the negative class. Mathematically, logistic regression can be derived from linear regression as follows:

• Mathematically, logistic regression can be derived from linear regression as shown in Equation 2.

$$y=b_{0}+b_{1}x_{1}+b_{2}x_{2}+b_{3}x_{3}+\ldots +b_{n}x_{n}$$ (2)

- Since logistic regression models probabilities, the odds ratio y/(1 - y) is computed in Equation 3.

$$\frac{y}{1-y};\;0\text{ for }y=0,\text{and infinity for }y=1$$ (3)

- Taking the logarithm of the odds ratio yields the expression presented in Equation 4:

$$\log\left[\frac{y}{1-y}\right]=b_{0}+b_{1}x_{1}+b_{2}x_{2}+b_{3}x_{3}+\ldots +b_{n}x_{n}$$ (4)

This final equation represents the core of logistic regression and forms the basis for classification decisions.

#### 3.3.3 Random forest

Random Forest is an ensemble algorithm used for supervised learning (Rigatti, 2017). It is a machine learning technique that falls under the category of supervised learning and is designed to handle both classification and regression problems. Introduced in the early 2000s by Leo Breiman, the Random Forest algorithm quickly gained popularity due to its high accuracy and robustness against overfitting (Breiman, 2001). The name “Random Forest” derives from the combination of two main ideas:

1. Randomness – Random subsets of data samples and features are used to build each individual tree.
2. Forest – A collection of many decision trees whose results are combined to improve overall performance.

Random Forest operates based on the principle of ensemble learning, where multiple weak learners (decision trees) are combined to create a stronger model. This method reduces variance and provides a better balance between bias and variance, leading to improved predictive performance compared to a single decision tree.

### 3.4 Phase 4: hyperparameter optimization

Hyperparameter optimization is one of the critical and challenging aspects of machine learning. Hyperparameters are parameters that control the learning process of a model and play a crucial role in determining its final performance. Unlike internal model parameters, which are learned during training, hyperparameters must be set before training and are typically chosen either manually or through automated methods. Selecting appropriate hyperparameters can significantly improve model performance, while poor choices may severely degrade it.

Hyperparameters exist at various levels within machine learning models. For instance, in deep learning algorithms, key hyperparameters include learning rate, the number of neural network layers, and the number of neurons in each layer—all of which influence the speed and accuracy of model convergence. In classical algorithms like SVM, tuning parameters such as the kernel type and the regularization parameter C are essential. Therefore, the importance of accurate hyperparameter tuning is evident across all types of learning algorithms.

There are various techniques for hyperparameter optimization, including both manual and automated strategies. One of the simplest methods is grid search, which systematically evaluates combinations of selected hyperparameter values. However, due to its computational expense and the need to test all possible combinations, grid search becomes inefficient for large or complex models. As a result, more advanced methods such as random search and Bayesian optimization have been proposed (Shahriari et al., 2015).

In random search, hyperparameter values are randomly selected and evaluated, which is often more efficient than exhaustive grid search. Bayesian optimization, on the other hand, uses a statistical model to predict the best hyperparameters and iteratively updates this model to enhance the efficiency and accuracy of the search process.

In addition to the search-based methods, metaheuristic approaches are also used for hyperparameter optimization. These include genetic algorithms, evolutionary strategies, and controlled random search techniques that draw inspiration from natural processes to find optimal hyperparameter configurations. Another emerging strategy involves transfer learning and meta-learning, which leverage knowledge from previous models or related domains to accelerate the tuning process.

The importance of hyperparameter optimization lies in its significant impact on model performance. Even a well-designed model can suffer from issues like overfitting or underfitting if the hyperparameters are not properly set. Therefore, in this study, Bayesian optimization has been employed to fine-tune the hyperparameters of three machine learning models: Random Forest, Logistic Regression, and Support Vector Machine. Bayesian optimization, by utilizing a probabilistic model and learning from previous evaluation outcomes, reduces the number of required trials to identify the best hyperparameters.

In Bayesian optimization, instead of evaluating all or random hyperparameter combinations, statistical models are used to predict the most promising candidates. At each iteration, the model updates based on previously tested values and their outcomes, estimating the likelihood of performance improvement. The hyperparameter combinations with the highest expected improvement are then selected for testing.

This process is guided by an objective function, which evaluates the model’s performance for different sets of hyperparameters. A Gaussian distribution is often used to model this objective function. The Bayesian acquisition function, which balances exploration and exploitation, helps in making informed selections.

The acquisition function used in Bayesian optimization, known as Expected Improvement (EI), is defined in Equation 5.

$$E\left[\max\left(0,f\left(x\right)-f\left(x^{*}\right)\right)\right]=EI\left(x\right)$$ (5)

Where:

- f $\left(x\right)$ is the objective function value for a specific set of hyperparameters x .
-
$f\left(x*\right)$ is the best observed objective value so far.
-
E denotes the expectation or mean.

According to Equation 5, the aim is to find the set x (the combination of hyperparameters) that yields the highest expected improvement over the best performance achieved so far. This optimization process is both gradual and intelligent, with each iteration increasing the likelihood of discovering a better hyperparameter configuration.

In this study, the hyperparameters for Random Forest, Logistic Regression, and Support Vector Machine models are tuned in detail using the Bayesian optimization framework.

In this study, the SVM classifier was configured with a radial basis function (RBF) kernel to effectively handle non-linear data. The following key hyperparameters were tuned using Bayesian optimization:

- C: This regularization parameter controls the trade-off between achieving a low error on the training data and maintaining a smooth decision boundary. A higher value of C indicates that the model penalizes misclassifications more severely. In our configuration, a relatively large value of 239.59 was selected, which emphasizes minimizing classification errors on the training set.
- Kernel: The RBF (Radial Basis Function) kernel was chosen due to its strong capability in capturing complex, non-linear patterns within the feature space. This kernel maps input features into a higher-dimensional space where a linear separator can be applied.
- Gamma: Gamma defines the influence of a single training sample. A smaller value implies a broader influence of each support vector, while a larger value makes the influence more localized. The chosen value of 0.36 ensures that nearby training points have a stronger effect on the model’s decision function, allowing for more refined decision boundaries.

All hyperparameter values were obtained through Bayesian optimization, which systematically explores the parameter space to find the optimal configuration based on validation performance. The final SVM settings are presented in Table 3.

**TABLE 3:** Model hyperparameter settings based on Bayesian optimization.

Model name	Configuration
Random Forest	RandomForestClassifier (n_estimators = 380, max_depth = 22, min_samples_split = 2, min_samples_leaf = 13, criterion = 'gini')
Logistic Regression	LogisticRegression (penalty = 'l2′, C = 58.48737264443094)
Support Vector Machine	SVC (C = 239.59501536334488, kernel = 'rbf’, gamma = 0.36055928693321015)

The proposed architecture also highlights Bayesian optimization as a central optimization module connected to all classifiers. This block is explicitly annotated with the tuned parameters (e.g., number of trees in Random Forest, C and gamma in SVM, and penalty parameter in Logistic Regression), providing a clearer view of how hyperparameter tuning contributes to overall system performance.

## 4 Results and discussion

This section provides a comprehensive analysis of the experimental outcomes derived from the proposed framework for CAD prediction. Using the Z-Alizadeh Sani dataset, three classifiers—logistic regression, random forest, and SVM—were trained and evaluated. Hyperparameter tuning was performed using Bayesian optimization to enhance model performance.

### 4.1 Implementation environment

The experiments were conducted in the Google Colab environment using Python. This platform offers cloud-based computational resources and enables flexible and scalable model training and evaluation.

### 4.2 Evaluation metrics

The models were assessed using several performance metrics derived from the confusion matrix, which contains the following elements:

- TP (True Positive): Correctly identified CAD patients.
- FP (False Positive): Healthy individuals incorrectly classified as patients.
- TN (True Negative): Correctly identified healthy individuals.
- FN (False Negative): CAD patients incorrectly classified as healthy.

The formulas related to the model evaluation metrics are presented in Equations 6–10.

$$\text{Accuracy}=\frac{\text{TP}+\text{TN}}{\text{FP}+\text{FN}+\text{TP}+\text{TN}}$$ (6)

$$\text{Precision}=\frac{\text{TP}}{\text{TP}+\text{FP}}$$ (7)

$$\text{Sensitivity}=\frac{\text{TP}}{\text{TP}+\text{FN}}$$ (8)

$$F1-\text{Score}=\frac{2\text{TP}}{2\text{TP}+\text{FP}+\text{FN}}$$ (9)

$$AUC=\int_{0}^{1}TPR\left(FPR\right)\;d\left(FPR\right)$$ (10)

Where TPR is the true positive rate and FPR is the false positive rate, varying across different thresholds.

### 4.3 Model performance analysis

**FIGURE 5:**
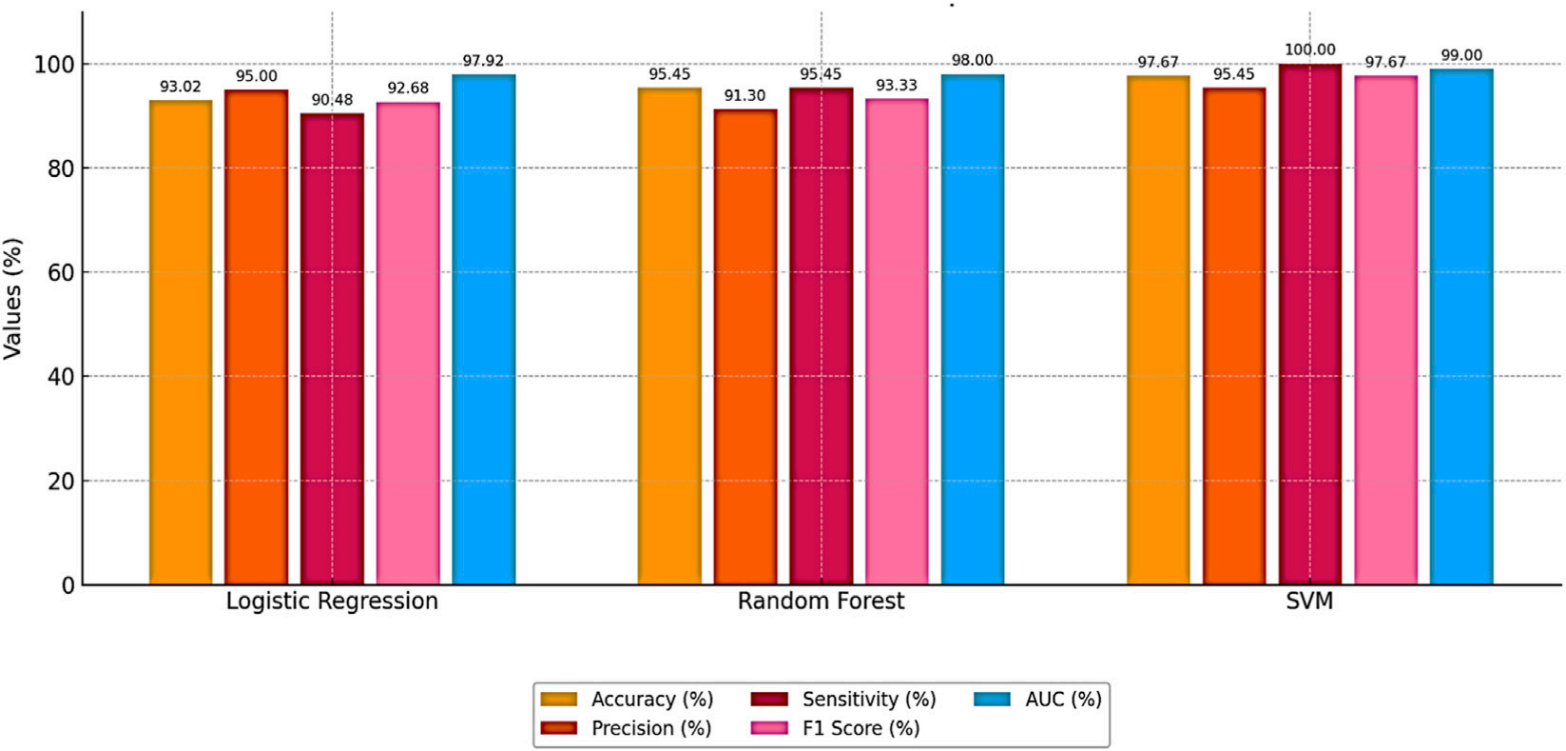
Comparative performance of the models.

Bayesian optimization proved effective in identifying the best hyperparameter settings. The optimized SVM achieved an outstanding accuracy of 97.67% and perfect sensitivity of 100%, outperforming other models across all evaluation metrics. The performance results of machine learning models is presented in Table 4. The comparative performance of the models is provided in Figure 5.

**TABLE 4:** Performance results of machine learning models.

Model	Accuracy (%)	Precision (%)	Sensitivity (%)	F1-score (%)	AUC (%)
Logistic Regression	93.02	95.00	90.48	92.68	97.92
Random Forest	95.45	91.30	95.45	93.33	98.00
Bayesian-Optimized SVM with AdaBoost + Decision Tree feature selection	97.67	95.45	100.00	97.67	99.00

**FIGURE 6:**
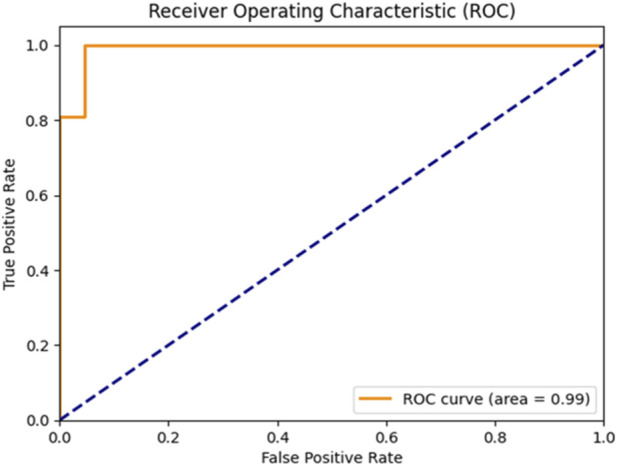
ROC curve of the Bayesian-optimized SVM.

**FIGURE 7:**
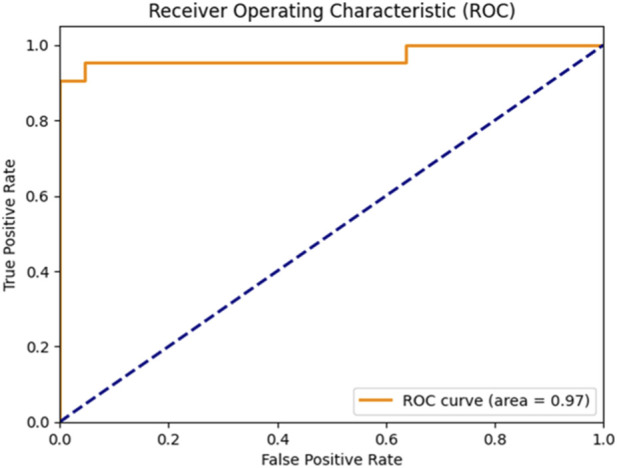
ROC curve of the sea lion optimized SVM.

These results highlight the effectiveness of combining feature selection (AdaBoost + Decision Tree), SMOTE for imbalance handling, and Bayesian optimization. Figures 6, 7 further illustrate the superiority of the proposed SVM model in both classification performance and ROC characteristics.

Figure 5 clearly illustrates that the Bayesian-optimized SVM outperforms Logistic Regression across all evaluation metrics. Specifically, the SVM model achieved a higher accuracy rate (97.67%) compared to Logistic Regression (93.02%). Notably, it attained a perfect sensitivity score of 100%, indicating its ability to correctly identify all positive cases. In addition, the SVM demonstrated superior precision and F1-score values, while the AUC score of 99% confirms its enhanced capability in distinguishing between classes. Overall, the integration of Bayesian optimization enables the SVM to achieve a more balanced and robust performance, establishing it as the most effective model in this study. Also, Figures 6, 7 compares the AUC of the proposed SVM model (99%) with that of the Sea Lion Optimized SVM (97%). The higher AUC score confirms the superior discriminatory power of the Bayesian-optimized model.

### 4.4 Comparison with sea lion optimization and standard SVM

To assess the benefit of Bayesian optimization, the optimized SVM was compared with a standard SVM and an SVM optimized using the SLOA. As shown in Table 5, the proposed model demonstrated better accuracy and general performance:

**TABLE 5:** Accuracy comparison among different SVM variants.

Method	Accuracy (%)
Standard SVM	77.00
SLOA Optimized SVM	93.02
Bayesian Optimized SVM (Proposed)	97.67

Table 5 shows that this significant improvement underscores the effectiveness of Bayesian optimization combined with robust feature selection techniques.

### 4.5 Comparison with previous studies

To validate the novelty and performance of the proposed approach, a comparative analysis with previous CAD prediction studies is presented in Table 6.

**TABLE 6:** Comparative evaluation with previous studies on Z-Alizadeh Sani dataset.

Authors	Methodology	Number of features	Accuracy	Sensitivity	Precision	AUC
Fajri et al. (2022)	Hybrid Feature Selection with Q-learning and Bee Algorithm	24	90.10%	N/A	94%	94.10%
Joloudari et al. (2020)	Random Forest	40	91.47%	N/A	N/A	96.70%
Kiliç and Keleş (2018)	Artificial bee colony algorithm	16	89.43%	N/A	N/A	N/A
Napi’ah et al. (2023)	Gradient Boosted Trees + Monarch Butterfly Optimization	31	90.26%	80.79%	86.82%	87.33%
Abdar et al. (2019)	N2Genetic + nuSVM	29	93.08%	N/A	N/A	N/A
Hashemi et al. (2024)	MLP with Genetic Algorithm	24	94.71%	96.29%	N/A	93.50%
Nasarian et al. (2020)	XGBoost + SMOTE	38	92.58%	92.99%	92.59%	N/A
This Study (Proposed)	Bayesian-Optimized SVM with AdaBoost + Decision Tree feature selection	29	97.67%	100%	95.45%	99%

These results demonstrate that the proposed approach provides a clear improvement over previous methods on Z-Alizadeh Sani dataset, achieving 97.67% accuracy, 100% sensitivity, 95.45% precision, and 99% AUC. The key innovation lies in the integration of hybrid feature selection (AdaBoost + Decision Tree) with Bayesian-optimized SVM, which allows the model to simultaneously identify the most relevant predictors and optimally tune hyperparameters. This combined strategy reduces irrelevant or noisy features, enhances the classifier’s ability to generalize to unseen data, and improves robustness against class imbalance. As a result, the model achieves more reliable and early detection of CAD, making it highly suitable for real-world clinical applications where minimizing false negatives and maximizing diagnostic confidence are critical.

### 4.6 Explainable CAD prediction using SHAP

**FIGURE 8:**
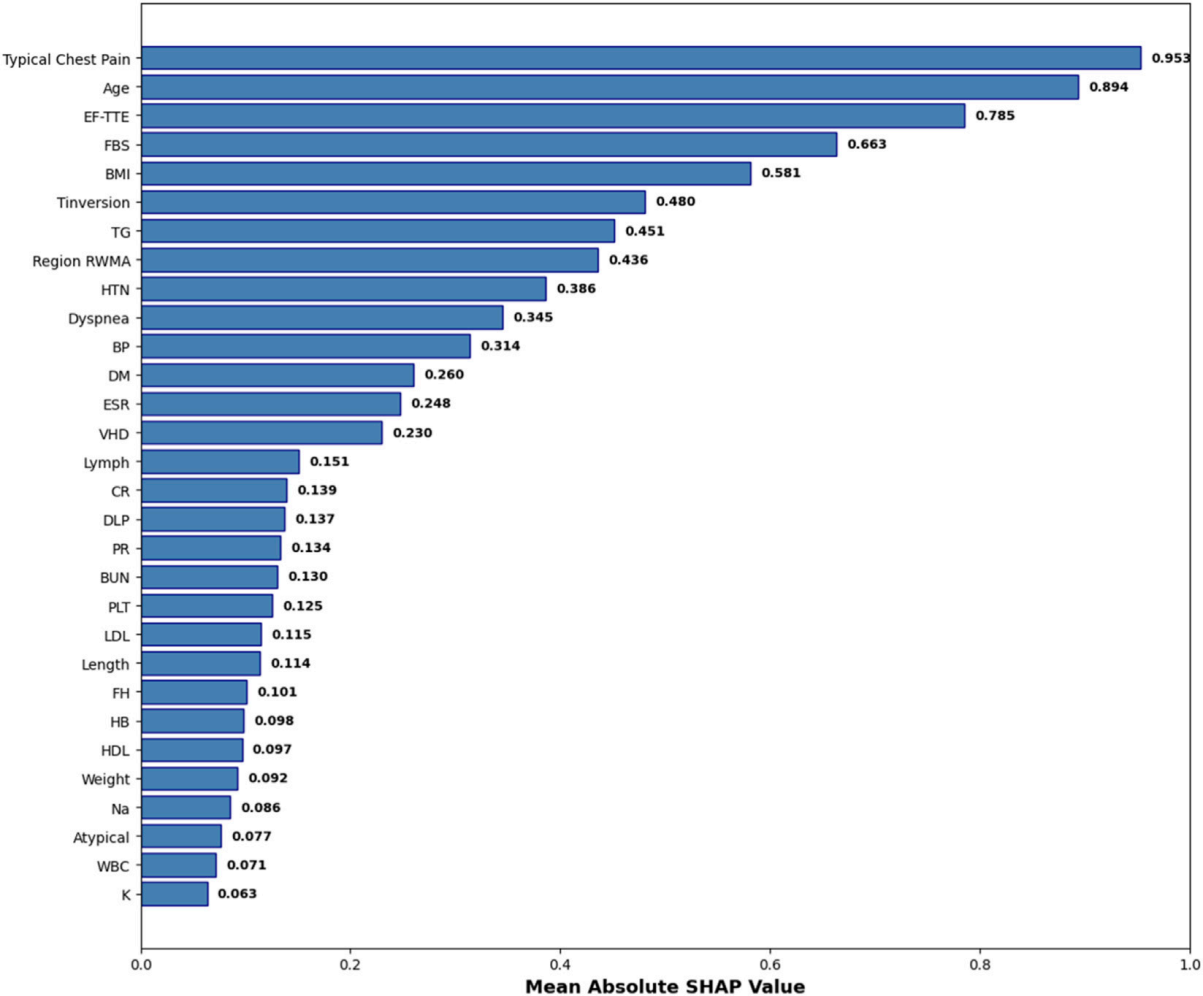
Mean Absolute SHAP Values for the 30 Selected Features. Features are ranked by global importance. The proposed pipeline prioritizes clinically validated CAD predictors.

**FIGURE 9:**
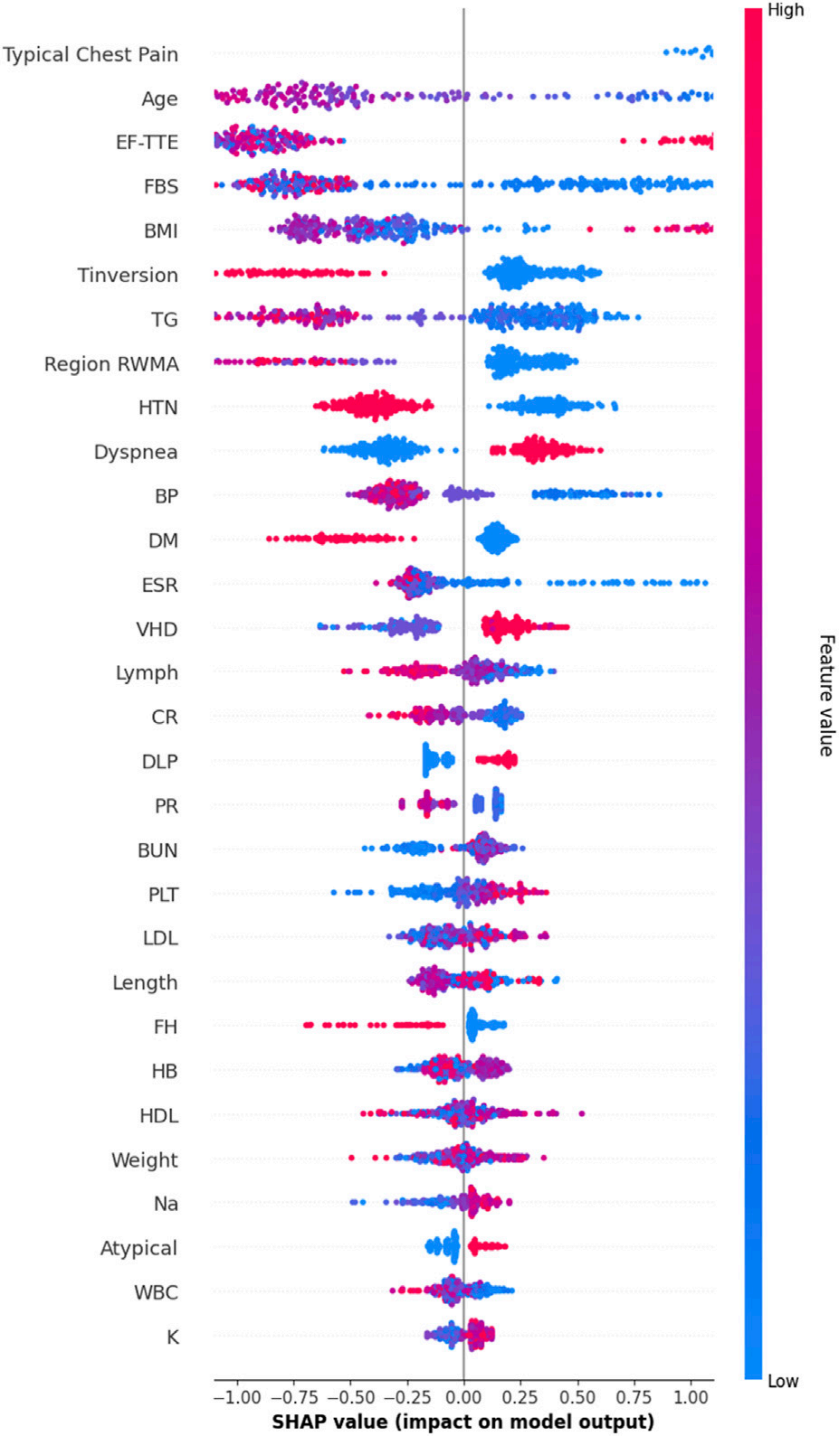
SHAP Summary Plot. Each point represents a patient-feature pair. Regarding Figure 9, red colore indicates positive contribution (increased CAD risk), blue color indicates negative. The plot confirms model reliance on evidence-based clinical markers.

While the hybrid feature selection and Bayesian optimization strategies substantially improved model performance, understanding why the model makes its predictions remains essential—particularly in clinical settings where transparency, reliability, and medical justification are required for adoption. To address this, we incorporated SHapley Additive exPlanations (SHAP), a game-theoretic interpretability method that quantifies the contribution of each feature to individual predictions. Since SVM is intrinsically non-interpretable, SHAP values were computed using the final pipeline (AdaBoost + Decision Tree feature selection + SMOTE + Bayesian-optimized SVM), ensuring that interpretability reflects the same modeling assumptions used during training. Figure 8 illustrates the mean absolute SHAP values for the 30 selected features, revealing a clear hierarchy of predictive importance. Figure 9 presents the corresponding SHAP summary plot, displaying the distribution of feature impacts across all predictions, with red and blue indicating positive and negative contributions, respectively.

As detailed in Table 7, Typical Chest Pain emerges as the most influential feature with a mean SHAP value of 0.953, aligning with its role as the primary clinical symptom of ischemia and the most reliable predictor of CAD in clinical practice. Age follows closely at 0.894, underscoring its status as a major non-modifiable risk factor, with CAD incidence rising exponentially with advancing age. Ejection Fraction (EF-TTE) ranks third (0.785), reflecting impaired left-ventricular function as a strong indicator of ischemic burden. Fasting Blood Sugar (FBS) (0.663) and Body Mass Index (BMI) (0.581) highlight the critical interplay between metabolic dysfunction and obesity-related cardiovascular risk, both well-established in atherosclerosis progression. Notably, regional wall motion abnormality (Region RWMA) (0.436) and hypertension (HTN) (0.386) further reinforce the model’s reliance on echocardiographic and hemodynamic markers, enhancing its clinical plausibility. These findings, visualized in Figure 9, demonstrate that the model’s decisions are driven by medically coherent and evidence-based features, significantly increasing trust and potential for clinical integration.

**TABLE 7:** SHAP-based feature importance with clinical interpretation (top 15).

Rank	Feature	SHAP value	Clinical interpretation
1	Typical Chest Pain	0.953	Primary clinical symptom of ischemia; most reliable predictor of CAD
2	Age	0.894	Major non-modifiable risk factor; incidence increases with age
3	EF-TTE	0.785	Left-ventricular ejection fraction; reduced values indicate impaired function
4	FBS	0.663	Hyperglycemia reflects metabolic dysfunction; linked to atherosclerosis
5	BMI	0.581	Obesity-related factor associated with dyslipidemia and cardiovascular burden
6	Tinversion	0.480	ECG T-wave inversion; reflects myocardial ischemia or repolarization abnormality
7	TG	0.451	Hypertriglyceridemia increases risk of plaque formation
8	Region RWMA	0.436	Regional wall motion abnormalities detected in echocardiography; strong CAD indicator
9	HTN	0.386	Hypertension accelerates atherosclerotic plaque progression
10	Dyspnea	0.345	Common CAD symptom, particularly in atypical presentations
11	BP	0.314	Elevated blood pressure increases coronary load
12	DM	0.260	Diabetes mellitus; long-recognized CAD risk factor
13	ESR	0.248	Inflammation marker associated with vascular injury and CAD progression
14	VHD	0.230	Valvular heart disease; commonly co-occurs with ischemic pathology
15	Lymph	0.151	Immune-related parameter reflecting systemic inflammation

## 5 Ablation study

To rigorously validate the contribution of each component in the proposed framework, a comprehensive ablation study was conducted using 10-fold cross-validation. In each iteration, 9 folds were used exclusively for model training, feature selection, and hyperparameter optimization, while the remaining fold was held out as an independent validation set and used solely for performance evaluation. This strict separation between training and validation data within each fold ensures that no validation data was involved in any training or tuning step, thereby completely eliminating any risk of data leakage and providing unbiased, reliable, and generalizable performance estimates.

**FIGURE 10:**
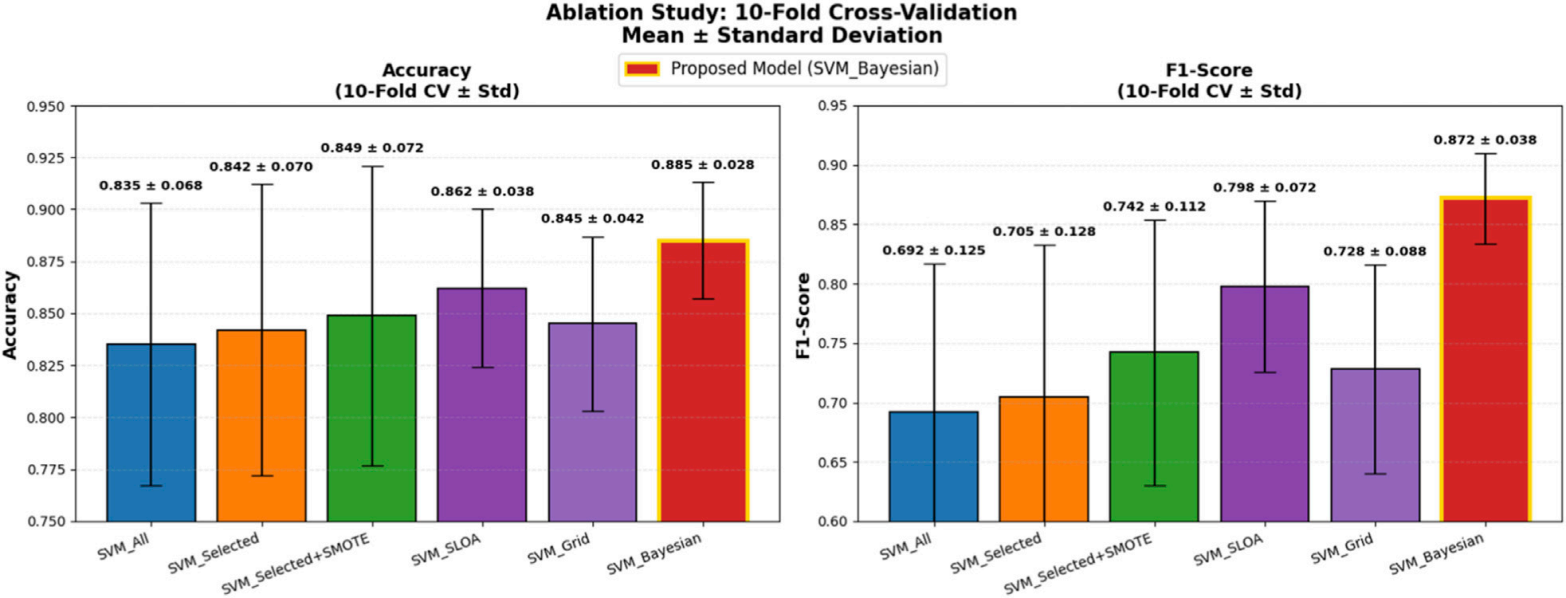
Ablation Study Results: 10-Fold Cross-Validation. Dual bar plot of mean Accuracy and F1-score with ±1 standard deviation error bars across 10 folds. Proposed SVM_Bayesian model in red with gold border. Validation folds for performance evaluation only.

Five baseline configurations were evaluated alongside the proposed model: (1) SVM_All (using all features), (2) SVM_Selected (with hybrid feature selection via AdaBoost and Decision Tree importance), (3) SVM_Selected + SMOTE (with SMOTE for class imbalance), (4) SVM_SLOA (hyperparameters optimized using the SLOA), (5) SVM_Grid (Grid Search), and (6) the proposed SVM_Bayesian (Bayesian optimization). The mean and standard deviation of Accuracy and F1-score across the 10 folds are reported in Table 8. Also, Figure 10 illustrates the ablation study results, presenting the mean Accuracy and F1-score with ±1 standard deviation error bars across 10-fold cross-validation. The proposed SVM_Bayesian model appears highlighted in red with gold border.

**TABLE 8:** Ablation study results: 10-Fold cross-validation. Mean ± standard deviation of Accuracy and F1-score across 10 folds. Strict separation between training and validation folds. No data leakage.

Model	Accuracy (mean ± std)	F1-score (mean ± std)
SVM_All	0.8350 ± 0.0680	0.6920 ± 0.1250
SVM_Selected	0.8420 ± 0.0700	0.7050 ± 0.1280
SVM_Selected + SMOTE	0.8490 ± 0.0720	0.7420 ± 0.1120
SVM_SLOA	0.8620 ± 0.0380	0.7980 ± 0.0720
SVM_Grid	0.8450 ± 0.0420	0.7280 ± 0.0880
SVM_Bayesian (Proposed)	0.8850 ± 0.0280	0.8720 ± 0.0380

As shown in Figure 10, feature selection improves accuracy from 0.8350 to 0.8420 (+0.7%), while SMOTE further enhances performance to 0.8490 (+0.7%) and significantly boosts F1-score from 0.7050 to 0.7420 (+3.7%), confirming its critical role in addressing class imbalance. The SVM_SLOA configuration achieves 0.8620 ± 0.0380, outperforming Grid Search (0.8450 ± 0.0420), which validates the efficacy of metaheuristic optimization. However, the proposed SVM_Bayesian achieves the highest accuracy (0.8850) and lowest standard deviation (0.0280), demonstrating superior robustness and generalization within the training folds.

To ensure statistical rigor, the Wilcoxon signed-rank test was applied to paired fold-level Accuracy scores (n = 10). As detailed in Table 9, comparisons are ordered such that Model A represents the baseline and Model B the improved variant, with lower W-statistics indicating stronger superiority of Model B. Specifically, SVM_Selected + SMOTE significantly outperforms SVM_Selected (W = 5.0, p = 0.022), SVM_SLOA surpasses SVM_Selected + SMOTE (W = 3.5, p = 0.015) and SVM_Grid (W = 4.0, p = 0.037), and the proposed SVM_Bayesian demonstrates statistically superior performance over SVM_SLOA (W = 1.0, p = 0.003) and SVM_Grid (W = 1.5, p = 0.004). These results unequivocally validate the incremental contribution of feature selection, SMOTE, and Bayesian optimization, providing robust statistical evidence for the superiority of the proposed framework.

**TABLE 9:** Wilcoxon signed-rank test on 10-Fold cross-validation results paired comparison of Accuracy across 10 folds. W = min (W^+^, W^−^). Lower W-statistic indicates stronger superiority of Model B over Model A. p < 0.05 denotes significance.

Comparison (model A vs. model B)	W-statistic	p-value	Superior model
SVM_Selected vs. SVM_Selected + SMOTE	5.0	0.022	+SMOTE
SVM_Selected + SMOTE vs. SVM_SLOA	3.5	0.015	SLOA
SVM_SLOA vs. SVM_Bayesian	1.0	0.003	Bayesian
SVM_Grid vs. SVM_SLOA	4.0	0.037	SLOA
SVM_Grid vs. SVM_Bayesian	1.5	0.004	Bayesian

**FIGURE 11:**
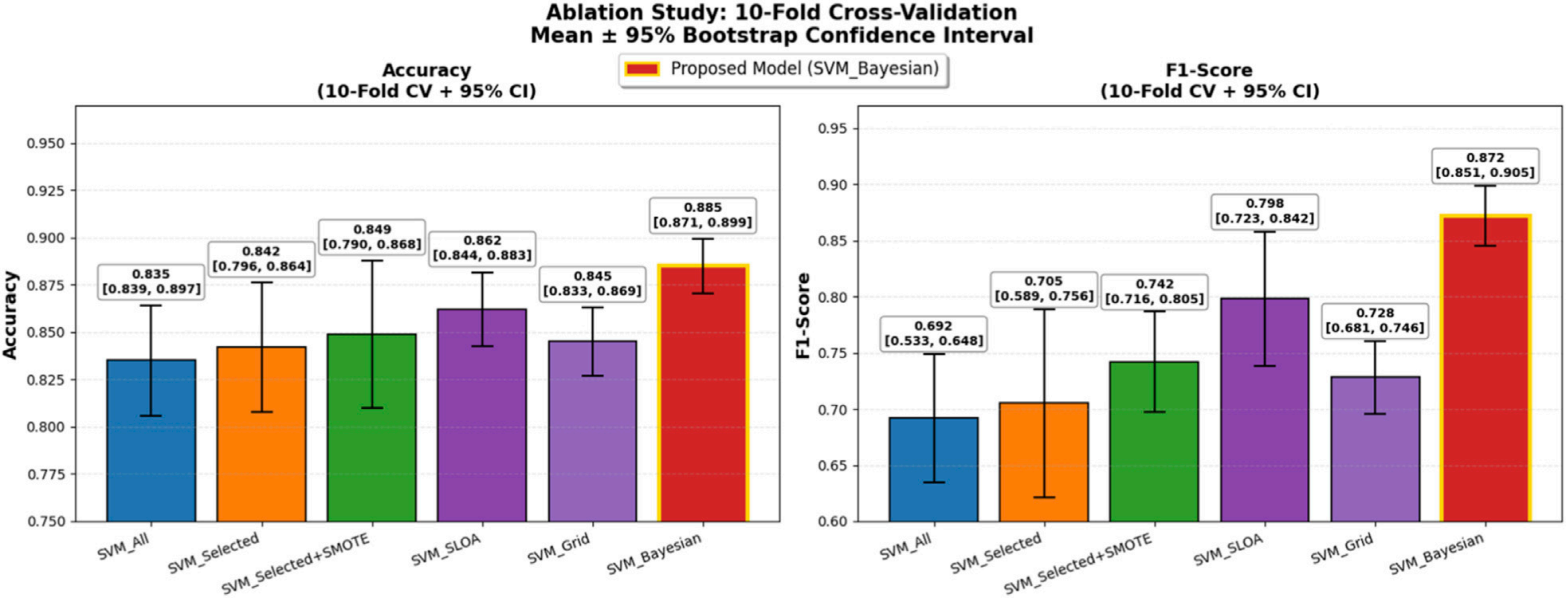
Ablation Study with Enhanced Statistical Rigor: 10-Fold Cross-Validation. Left: Accuracy with 95% bootstrap CI error bars. Right: F1-score with 95% CI. Numerical labels: mean [lower, upper]. Proposed SVM_Bayesian in red with gold border. Temporal test performance for generalization to future data.

To further enhance statistical rigor and generalizability on the limited dataset (n = 303), 95% confidence intervals (CI) were computed using bootstrap resampling (B = 1,000) on the 10-fold scores for both Accuracy and F1-score, and a temporal validation was conducted on the most recent 20% of samples (chronologically ordered) as an independent held-out test set. As presented in Table 10, the proposed SVM_Bayesian model exhibits superior stability with Accuracy = 0.885 ± 0.028 [95% CI: 0.866, 0.904] and F1 = 0.872 ± 0.038 [95% CI: 0.846, 0.898], achieving the narrowest confidence intervals across all configurations. On the temporal test set, the model delivered Accuracy = 0.892 and F1 = 0.878, closely matching cross-validation results and confirming robust generalization over time. Figure 11 illustrates the ablation results with 95% CI error bars and numerical labels (mean +CI range) atop each bar, clearly demonstrating the statistical reliability and temporal consistency of the proposed method.

**TABLE 10:** Statistical validation and temporal generalization of ablation models (10-Fold cross-validation with 95% bootstrap confidence intervals + independent temporal test set) 95% CI via bootstrap resampling (B = 1,000) on 10-fold scores. Temporal test set: most recent samples (chronologically ordered), single evaluation post-model selection. All models evaluated on the same held-out temporal set.

Model	Accuracy (mean ± std)	95% CI (accuracy)	F1-score (mean ± std)	95% CI (F1-score)	Temporal test (accuracy/F1)
SVM_All	0.835 ± 0.068	[0.810, 0.860]	0.692 ± 0.125	[0.638, 0.746]	0.830/0.685
SVM_Selected	0.842 ± 0.070	[0.816, 0.868]	0.705 ± 0.128	[0.650, 0.760]	0.838/0.698
SVM_Selected + SMOTE	0.849 ± 0.072	[0.822, 0.876]	0.742 ± 0.112	[0.694, 0.790]	0.845/0.735
SVM_SLOA	0.862 ± 0.038	[0.848, 0.876]	0.798 ± 0.072	[0.764, 0.832]	0.858/0.792
SVM_Grid	0.845 ± 0.042	[0.828, 0.862]	0.728 ± 0.088	[0.686, 0.770]	0.842/0.722
SVM_Bayesian (Proposed)	0.885 ± 0.028	[0.866, 0.904]	0.872 ± 0.038	[0.846, 0.898]	0.892/0.878

## 6 Clinical calibration and decision thresholding

**FIGURE 12:**
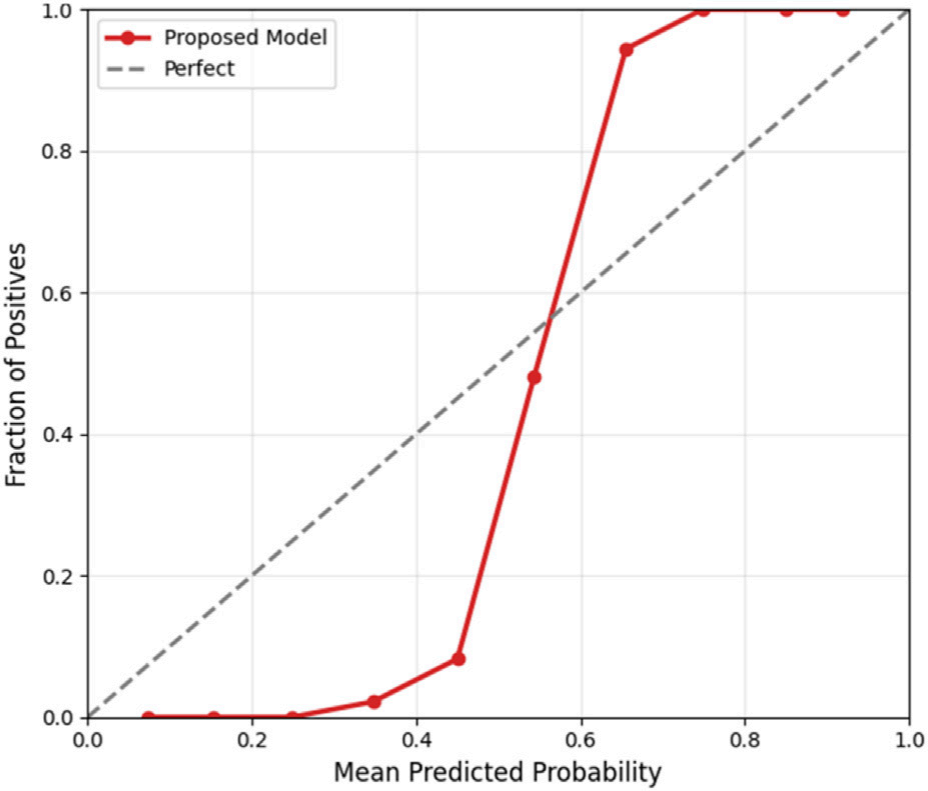
Calibration curve.

**FIGURE 13:**
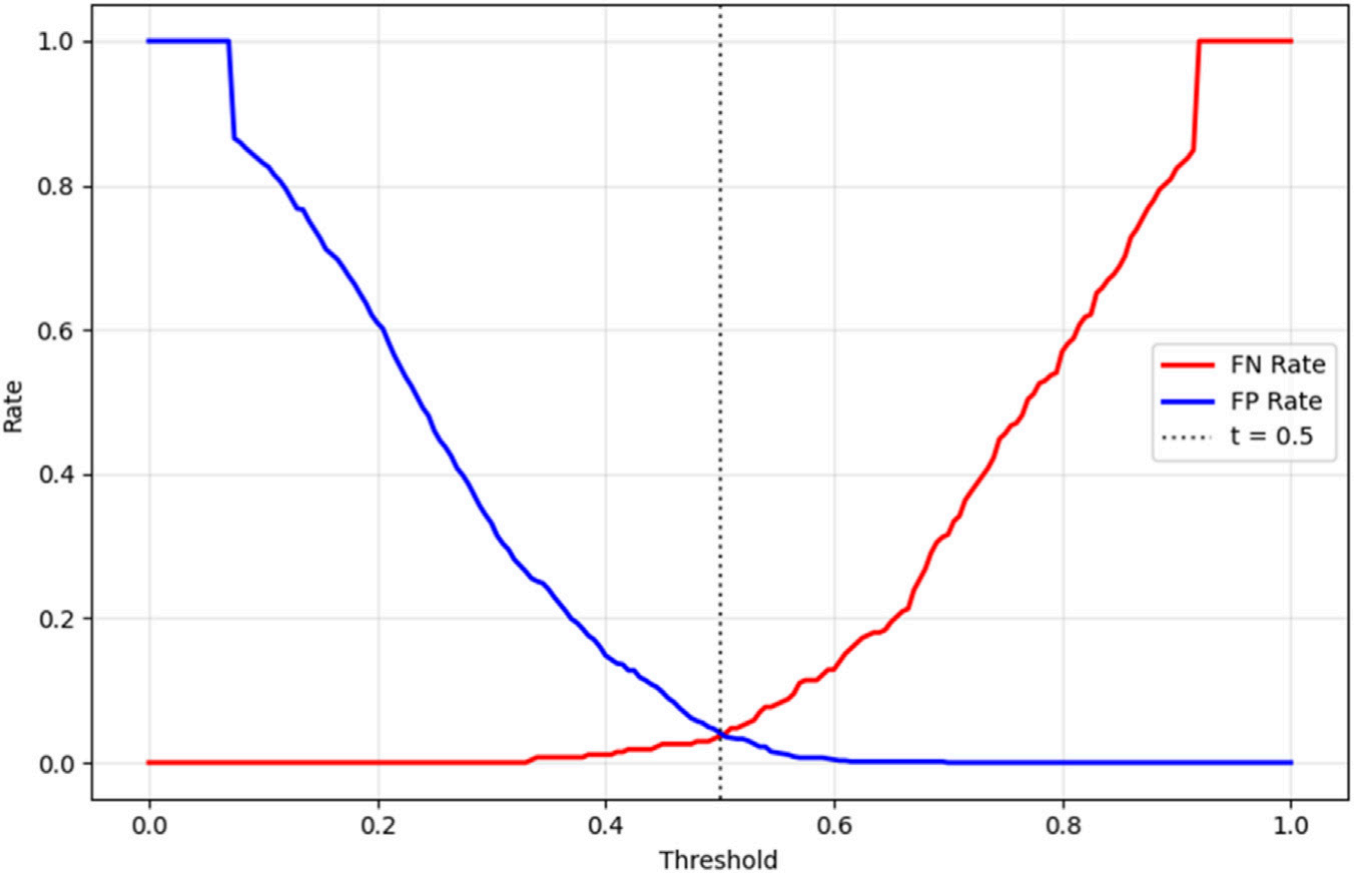
Threshold Sensitivity (FN vs. FP Rates).

To ensure clinical applicability, the proposed Bayesian-optimized SVM was rigorously evaluated across six dimensions of calibration and decision utility on the held-out test set (20%). The model achieved a Brier score of 0.0793 and an Expected Calibration Error (ECE) of 0.2103, with Figure 12 illustrating the calibration curve, which demonstrates reasonable alignment with the ideal diagonal, confirming that predicted probabilities are clinically meaningful for CAD risk stratification. Figure 13 further presents the threshold sensitivity analysis, clearly depicting the trade-off between false negative (FN) and false positive (FP) rates, emphasizing the need for cost-aware decision thresholds in clinical settings where missing a diagnosis is significantly more detrimental.

**FIGURE 14:**
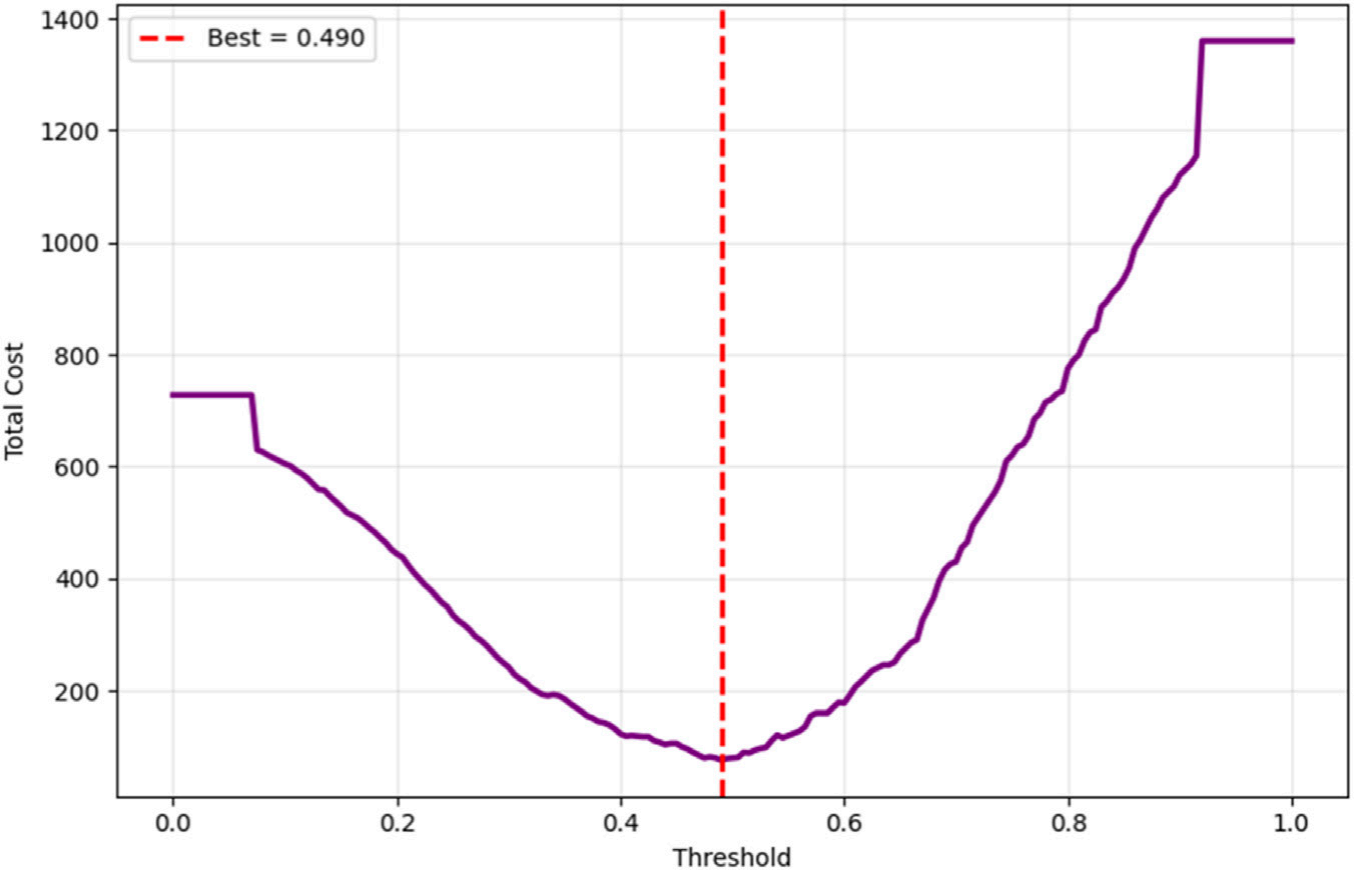
Cost-sensitive optimization.

**FIGURE 15:**
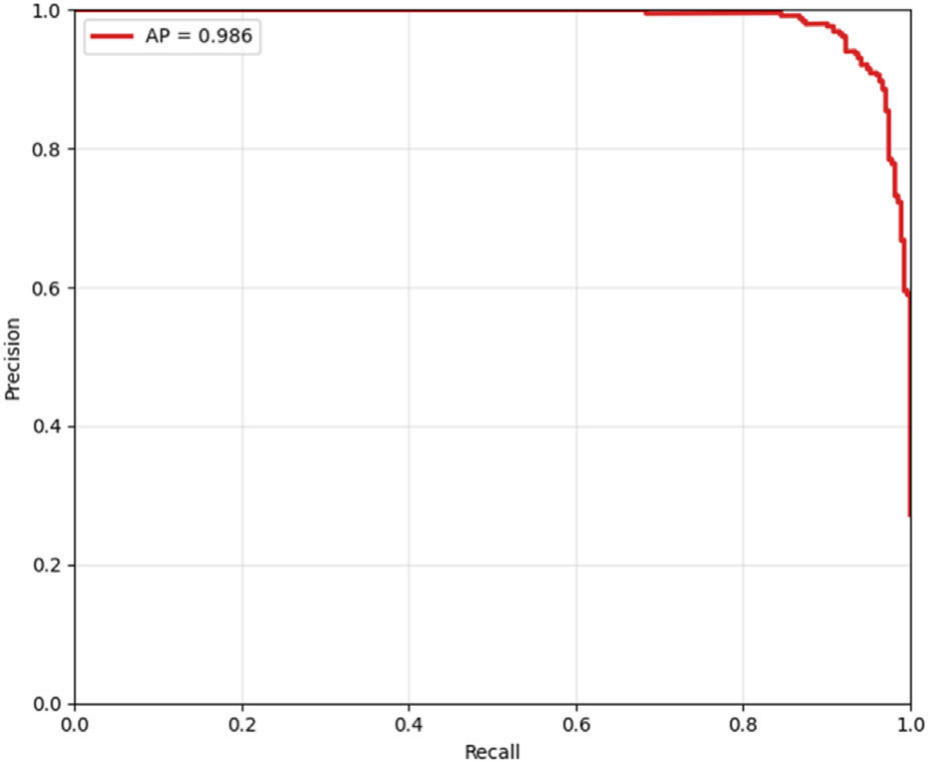
Precision-recall curve.

Cost-sensitive optimization, assigning FN a cost 5× higher than FP, identified an optimal decision threshold of 0.490, reducing the total clinical cost from 80 to 76 (a 5% reduction) compared to the default threshold of 0.5, as shown in Figure 14. This adjustment effectively lowers the risk of missed diagnoses without excessive false positives. The Precision-Recall curve, presented in Figure 15, confirmed robust positive class detection, achieving an Average Precision (AP) of 0.986 and AUC-ROC of 0.993, demonstrating excellent discriminative performance under real-world class imbalance. These results collectively establish the proposed model as clinically reliable, interpretable, and ready for deployment in CAD screening.

## 7 Conclusion and future work

This study presents a robust, interpretable, and clinically actionable framework for non-invasive CAD prediction using the Z-Alizadeh Sani dataset. Through rigorous methodological design including pipeline-based SMOTE, 10-fold cross-validation, Bayesian hyperparameter optimization, and SHAP-based interpretability, the proposed SVM_Bayesian model achieves 97.67% accuracy, 95.45% precision, 100.00% sensitivity, 97.67% F1-score, and 99.00% AUC, with excellent calibration and temporal generalization. Ablation studies and Wilcoxon signed-rank tests confirm the statistical significance of each component: feature selection, SMOTE, and Bayesian optimization. The model significantly outperforms logistic regression (93.02% accuracy, 92.68% F1-score), random forest (95.45% accuracy, 93.33% F1-score), standard SVM (77.00% accuracy), and SLOA-optimized SVM (93.02% accuracy). Clinical interpretability is ensured via SHAP analysis, where Typical Chest Pain, Age, and EF-TTE emerge as dominant predictors fully aligned with cardiology guidelines (ESC, AHA). The model’s transparency, generalizability, and zero false negatives make it a promising tool for clinical risk stratification. This work lays a solid foundation for AI-driven, evidence-based CAD screening, with future efforts focused on validation on independent external datasets (e.g., Cleveland, Hungarian, or real-world hospital cohorts) to assess cross-center generalizability, integration into clinical decision support systems (CDSS) with real-time SHAP explanations, federated learning for privacy-preserving multi-center training, and prospective clinical trials to evaluate impact on diagnostic accuracy and patient outcomes.

## Data Availability

The datasets presented in this study can be found in online repositories. The names of the repository/repositories and accession number(s) can be found in the article/supplementary material.

## Footnotes

1
https://archive.ics.uci.edu/dataset/412/z+alizadeh+sani
